# Northern and American Lancet

**Published:** 1851-09

**Authors:** 

**Affiliations:** Late Lecturer on Anatomy, in the School of Medicine of Montreal, Canada.


*Northern and American Lancet.* Edited by HORACE NELSON, M. D., late Lecturer on Anatomy, in the School of Medicine of Montreal, Canada.

The *Northern Lancet*, published at Plattsburg, N. Y., is a monthly of fifty-two pages, printed at the low rate of one dollar *per annum.* The worth of this periodical is not to be measured by its price. The editor, Dr. Nelson, is a vigorous writer, and does not hesitate to speak his mind, plainly and independently, on all subjects pertaining to the honor of the medical profession. In addition to the monthly, he has lately added a supplementary publication of eight pages, which appears weekly, under the title of the *American Lancet.* The circulation of these works, as would be expected, is extensive, sufficiently so, as we trust, to meet the views of the enterprising editor, who has our best wishes for the success of his efforts in behalf of American Medical periodical literature. We perceive that Dr. Nelson has lately been appointed Professor of Anatomy in the St. Lawrence Med. School, a new institution established in Montreal, Canada.

